# Short and Long-Term Effects of Compromised Birth Weight, Head Circumference, and Apgar Scores on Neuropsychological Development

**DOI:** 10.4172/2329-9525.1000127

**Published:** 2014-08-04

**Authors:** Stephanie B. Gampel, Yoko Nomura

**Affiliations:** 1 Albert Einstein College of Medicine, New York, USA; 2 Division of Child and Adolescent Psychiatry, Department of Psychiatry, Icahn School of Medicine at Mount Sinai, USA; 3 Department of Psychology, Queens College, CUNY, Flushing, New York, USA

**Keywords:** Low birth weight, Neuro cognitive functioning, Perinatal risk

## Abstract

**Background:**

Low birth weight (LBW, <2500 g) is an adverse perinatal risk that may reflect a poor intrauterine environment. While LBW has been a well-known predictor of physical, neurological, cognitive and psychological deficits later in life, minimal research has been done on small head circumference and low 5 minute Apgar scores, and their association with subsequent developmental abnormalities.

**Objective:**

The current study aims to demonstrate that small head circumference and low 5-minute Apgar scores are predictors for developmental abnormalities throughout childhood and later.

**Methods:**

Using a longitudinal design, 2,151 individuals' physical, neurological, and cognitive functioning in childhood, as well as psychological functioning in adulthood, was assessed as a function of three perinatal risk factors: LBW, small head circumference and low Apgar scores.

**Results:**

Similar to findings with LBW, small head circumference or a low Apgar score were associated with increased number of hospital visits (p<0.0001 and p=0.005 respectively) and neurological abnormalities (p<0.0001 and p=0.001 respectively) at age 1. Intelligence quotient (IQ) scores at ages 4 and 7 were significantly lower for those born with small head circumference (p<0.0001) or low Apgar scores (p=0.002). Finally, the incidence of anxiety in adulthood was significantly higher for those born with small head circumference (p=0.03) or low Apgar scores (p=0.004) compared to their counterpart.

**Conclusion:**

Small head circumference and low a Apgar score are predictors of later physical, neurological, cognitive and psychological abnormalities, and can complement LBW, a more frequently used perinatal risk factor, and thus be used to screen for future developmental deficits, together with LBW.

## Introduction

Low birth weight (LBW), defined as less than 2500 g, has been implicated as a predictor for subtle, but adverse, childhood neuro-cognitive functioning, including cognitive, neurological and psychological challenges [1-3]. Individuals born with LBW are shown to have poorer visuospatial skills and arithmetic abilities throughout their lives [4]. Other evidence reports that individuals born with LBW have an increased risk for hyperkinetic disorder and attentional problems both later in childhood [5-7] and adulthood [8]. More recently, LBW has been linked to autistic spectrum disorder [9,10]. However, in comparison to LBW, far less effort has been made to explore the potential usefulness of these additional perinatal risk factors, although these perinatal risk factors are routinely collected at birth in the current obstetric practices. Similarly, little research has been conducted to evaluate these measures' predictive ability of poor outcomes simultaneously throughout childhood, as well as their long-term influences upon adult functioning.

While not extensive, prior researchers have investigated the effects of small head circumference and low Apgar scores on various functioning of the offspring. For example, small head circumference at birth has been associated with increased vulnerability to the immature brain [11] lower verbal proficiency, visuospatial skills and arithmetic abilities in young adulthood,4, and an increased risk for depression and hypertension in adulthood [12,13]. Microcephaly is a predictor of significantly lower IQ at age [4,14] and patients with the disease scored significantly worse on cognitive tests at 56 months of age [15]. If microcephaly persists into infancy it is a predictor of lower IQ at age 814 and age 16. Low Apgar scores were also found to be associated with distressed conditions in utero, school performance, and various physical and psychiatric illnesses later in life [16-18]. Perinatal risk factors are also associated with neuropsychological deficits later in childhood. These include greater incidents of post-traumatic stress disorder and greater psychological stress and HPA-axis reactivity [19,20]. With technological advancement reducing mortality rates of LBW infants, it becomes more and more important to examine and detect long term negative consequences of perinatal abnormalities on neuro-developmental and functioning deficits. LBW, small head circumference and low Apgar scores together may demonstrate possible associations with cognitive, behavioral, emotional and medical functioning in children from infancy to early childhood.

The current study will examine the prevalence of abnormality in neurological development, cognitive functioning and academic achievement, medical problems, and language, hearing and speaking in childhood by the three perinatal risk factors, i.e, LBW, small head circumference, and low Apgar scores, simultaneously. LBW would serve as a reference, i.e, the established surrogate measure, in order to have a base of prediction with which we could compare the head circumference and Apgar scores predictability. Considering multiple indicators of poor intrauterine environment may also help identify infants at greater risk for neurobehavioral development and psychological symptoms in adulthood.

## Method

### Data source

Data come from part of the Johns Hopkins Collaborative Perinatal Study. Pregnant women receiving prenatal care at the perinatal clinic delivered their babies at Johns Hopkins Hospital during 1960-1964. Their children were followed until they were 8 years of age. Twenty-five years later, those offspring were re-contacted (mean age 31). Hardy et al. [21] for a full description of the overall study design and methodology.

### Subjects

Of the 2,694 2^nd^ generation subjects (G2) eligible for follow-up between 1992 and 1994, 2,344 (79.8%) G2 had information on the three perinatal problems (LBW, small head circumference, and low Apgar score) and an initial neurological examination at 4 months old. Between those who are included in the analysis (N=2,151) and those excluded (n=543), there is no major demographic differences except for the sex of the participants, where a greater proportion of male than female offspring was excluded. The frequency of missing data on neurobehavioral and cognitive function measures ascertained between ages 0 to 8 for dependent variables vary, ranging from 0% to 2.9%. 7.6 to 9.1% of speech, language, and hearing function at age 3, and 10.5% of the health care utility between 4 and 8 years old, and are missing.

Of those 2,151 cohorts, 1,540 were located 25 years later. Approximately 91% of the located sample (n=1403) provided their general psychological status. The frequency of missing data for controlling variables was negligible (less than 0.2%).

### Measures and assessments

#### Perinatal risk factors

Birth weight, head circumference, and 5 minute Apgar scores were recorded by a nurse observer in the delivery room at the birth of the infant. Mean (sd) of these three perinatal indicators are 3,024 g (561 g), 33.6 cm (4.0 cm), and 8.8 (1.2) respectively. These risk factors were dichotomized at the cut-off points, to create indicator variables for LBW (≤ 2,500 g), small head circumference (≤ 32 cm), and low Apgar score (<7), which are conventionally used at clinical setting.

#### Medical visits and hospitalization

The number of medical visits and the number of hospitalizations at ages 1, 4, and 8 year were reported based on caretakers' self-reports.

#### Neurological and non-neurological abnormality

Neurological abnormalities at ages 4 months, 1, and 7 years were evaluated through physical examination conducted by a pediatrician, with special training in neurology, or a pediatric neurologist. Neurological abnormalities included skull size and shape, spinal abnormalities, primary muscle disease, mental retardation, emotional and psychiatric disorders and detected squint. Non-neurological abnormalities included all conditions except neurological abnormities and minor acute upper respiratory infection. Based on observation of 116 items the child's neurological status was characterized as none (for normal functioning), suspect, or definite. Finally, the impression of the child's non-neurological status was characterized as none, minor, or questionable/definite.

### Neurodevelopmental and Cognitive Function Evaluation

#### Eight Month Assessment

Bayley Scales of Mental and Motor development [22] was used to evaluate the mental, fine-motor, gross-motor, and social-emotional development. Developmental tasks were scored pass or fail. The research child psychologist summarized the child's performance as advanced, normal, suspect or abnormal. The answer options of suspect or abnormal were given a value of 1 and advanced and normal 0. Mean (SD) age at the assessment is 8.1 (0.3) months.

#### Three Year Assessment

Speech, language, and hearing problems were assessed by a speech pathologist and audiologist, using The Three-Year Speech, Language, and Hearing Examination [23]. The examination consists of five areas (language reception, language expression, hearing, speech mechanism, and speech production). These five sub-areas and a summary measurement were used for each area rating the child's test performance as normal, suspect and abnormal. Normal was coded as 0 and suspect or abnormal was coded as 1.

#### Four Year Assessment

Developmental profiles were assessed by child psychologists in four areas: fine motor development, gross motor development, concept formation, and behavior. Each area was scored separately and the child's performance was indicated as “normal” or “suspect or abnormal.” Normal was coded as 0 and suspect or abnormal was coded as 1.

Fine motor development was measured by Wallin pegboard, coping forms, stringing beads, and porteus maze. Gross motor development was measured by line walk, hopping, and ball catch. Each developmental task was scored pass or fail and established the presence or absence of motor defects first. Concept formation was measured by Graham-Ernhart block sort test, which provided a significant discrimination between brain damaged and non-brain damaged preschoolers. The test consists of sorting materials based on size, shape and color.

Cognitive functioning was assessed using the Stanford-Binet IQ [24] and administered by a child psychologist when the child was within 3 months of age 4. IQ scores were standardized with a mean of 100 and a standard deviation of 15.

#### Seven to eight Year assessment

Child psychologists measured intelligence, visual motor coordination, and academic performance. Intelligence was measured using Wechsler Intelligence Scale for Children (WISC) at age 7 [25]. Standardized scores had a mean of 100 and a standard deviation of 15. Academic performance (i.e., reading, arithmetic, and spelling) at age 8 was measured by the Wide Range Achievement Test (WRAT).26 In view of the narrow age range of the sample at the time of testing; we used the raw scores for this analysis. Mean (SD) scores for the three areas were: Spelling: 30.8 (.20), Reading: 22.3 (.10), and Arithmetic: 19.0 (.08). Ranges were 0–76, 0–56, and 0–32 respectively.

#### Adult psychological functioning

Adult psychiatric status was measured using the General Health Questionnaire-28 (GHQ-28) [26,27]. Depression, social dysfunction, anxiety, and somatization were each assessed by means of 7 questions, with response options ranging from 1 (better than usual) to 4 (much worse than usual). Using the scoring method in the manual, a choice of 1 or 2 was re-coded as “0” and 3 or 4 as “1.” Based on the sum of the responses, dichotomous indices for each variable were created, with a score of 4 or more indicating the presence of each variable. Internal consistency of the GHQ, evaluated by testing split-half reliability, was 0.95 [28]. Compared to the three most commonly used instruments for identifying psychiatric illness (the Center for Epidemiological Studies Depression Scale, the Beck Depression Inventory, and the Hospital Anxiety and Depression Scale), the GHQ had higher sensitivity (92%) and specificity (90%) for identifying psychiatric illness [29,30].

#### Potential confounders

Socio-demographic confounders include mother's race and poverty level at delivery and child's sex. Poverty level represents the ratio of the mother's annualized income to the poverty level based on the Social Security Bulletin Annual Statistical Supplement [31]. Gestational duration at birth was also included in all multivariable analyses for a statistical adjustment. All confounders were based on a mother's self-report.

### Data analysis

First, to examine group differences by LBW, small head circumference, and low Apgar scores for rates of offspring problems at different times in infancy, childhood, and adulthood, univariate analyses were conducted using X2 tests for dichotomous outcomes and analysis of variance (ANOVA) for continuous outcomes. The univariate analyses were followed by multivariate analyses to adjust for potential confounders: logistic regression analysis was used for dichotomous outcomes and analysis of co-variance (ANCOVA) was used for continuous outcomes. Potential confounders were considered a priori and were included in the model as covariates for statistical adjustment. All reported p values are two-tailed.

## Results

### Characteristics of offspring

Among 2,151 offspring, 82.4% were Black, 17.94% were White, and 49.9% were female. Approximately a quarter of the mothers completed at least high school and approximately 30% had less than or equal to an 8th grade education. With regard to mothers' demographics, the mean (SD) number of progeny, age and individual income during the 1st trimester pregnancy were 3 (2.4), 24.9 (7.1) and $1,022 (515) respectively.

### The number of hospital admissions by perinatal risk factors

Table 1 shows mean (SD) numbers of hospital admissions by the three perinatal risk factors at three different periods in childhood (birth to 1 year, 1 to 4 year, and 4 to 8 year). All three were associated with greater numbers of hospital admissions during the period between birth and 1 year. However, as young participants grew, the differences in children by head circumferences (small vs. normal) and Apgar scores (low vs. normal) diminished, while LBW continued to be significantly associated with a higher number of hospital admissions at age 8.

### Overall neurological and non-neurological abnormalities by perinatal risk factors

Research pediatricians and pediatric neurologists who were blind to perinatal risk status, assessed development of the offspring in three categories (none, suspect and definite for neurological abnormality and none, minor, or questionable/definite for non-neurological abnormality) at ages 4 months, 1 year, and 7 years. Table 2 shows significant differences in the rates of neurological abnormality by perinatal risk factors. At 4 months and 1 year, the magnitude of association between any of the three perinatal risk factor and definite neurological abnormality is clinically significant, ranging from odds ratio (OR) of 3.9 to 5.9, indicating that there was an approximately 4 to 6-fold increased risk of definite neurological abnormality if offspring was born with LBW, small head circumference, and low Apgar scores. Except for LBW, the risk of definite neurological abnormality at age 7 years was significant, i.e., an approximately 2-fold increase risk among offspring with small head circumference and an over 3-fold increased risk among offspring with low Apgar scores.

LBW was associated with a 2-fold increased risk for definite non-neurological abnormality at 4 months and 1 year, but not with minor non-neurological abnormality, and the differences diminished by 7 years of age. There was a significant increased risk for questionable/definite non-neurological abnormality among offspring with small head circumference at 4 months. However, unlike with LBW, the risk for non-neurological abnormality diminished by 1 year of age. There was no notable increased risk of non-neurological abnormality (neither minor nor definite) with low Apgar scores.

### Cognitive neuropsychological development at age 8 months and 4 years by perinatal risk factors

Table 3 shows cognitive neuropsychological development of children with and without perinatal risks at 8 months old and 4 years old. At 8 months, LBW was associated with a 6-fold increased risk for mental abnormalities (p<0.0001), over a 4-fold increased risk fine motor abnormalities (p<0.0001), a 3-fold increased risk for gross-motor abnormalities (p<0.0001), and 2-fold increased risk for social and emotional development abnormalities (p<0.0001). Small head circumference was associated with a 3-fold increased risk for mental abnormalities (p<0.0001), fine motor abnormalities (p<0.0001), and gross motor abnormalities (p<0.0001) and a 2-fold increased risk for social & emotional abnormalities (p<0.0001). Low Apgar scores were also associated with an over 3-fold increased risk for mental abnormalities (p<0.0001), an over 2-fold increased risk for fine motor abnormalities (p<0.0001), gross motor (p<0.0001) and social & emotional (p=0.001) abnormalities.

At the 4 year neurobehavioral development assessment, with an exception of the association between low Apgar scores and concept formation, all of the three perinatal risk factors were associated with a smaller, but significant, increased risk for fine motor (adjusted odds ratio, AOR=1.6, 1.4, 1.5), gross motor (AOR=2.8, 1.9, 2.2), intelligence (AOR=1.8, 1.7, 1.9), and concept formation abnormalities (AOR=1.4, 1.0, 1.4)

### Cognitive and academic functioning

Table 4 shows cognitive functioning and academic functioning by the three perinatal risk statuses. With regards to cognitive function (ages 4 and 7) and academic functioning (age 8), all of the three perinatal risk factors we examined were consistently associated with lower IQ both at ages 4 and 7. Moreover, offspring with perinatal risk factors generally had lower academic functioning in reading, spelling, and arithmetic, while the greatest impairment was observed in arithmetic scores. There was, however, no association between low Apgar scores and spelling achievement (p=0.09).

### Language, hearing and speech impairment at 3 years old

Table 5 shows five areas of functioning, including: language perception, language expression, hearing, speech mechanism, and speech production, were measured at the 3 year old. All of the three perinatal risk factors were associated with moderate but elevated risks, ranging from 1.5 to 2.0, for language perception and language expression. Notably, only small head circumference shows significant increased risk, albeit small, for all five areas of language, hearing and speech functional impairment, while LBW was associated with increased risk for impairment in speech production. Global indices shows that only LBW (AOR=1.6, p=0.001) and small head circumference (AOR=2.0, p<0.0001), but not low Apgar scores, were associated with language, hearing and speech impairment.

### Psychological symptoms among adult offspring

Table 6 shows the risk for psychological functioning in adulthood by perinatal risk factors. LBW was associated with an approximately 2-fold increased risk for depression (p=0.05) and social dysfunction (p=0.005) and low Apgar scores was associated with an almost 2-fold increased risk for anxiety (p=0.004). It is notable that small head circumference was associated with significant increased risk for all of the psychological functioning assessed, including depression (AOR=2.6, p=0.01), social dysfunction (AOR=2.3, p=0.003), anxiety (AOR=1.4, p=0.03) and somatic symptoms (AOR=1.5, p=0.03), while the magnitude of the increase risk was sometimes less than 2-fold.

## Discussion

The current study evaluated the short- and long-term neuropsychological developmental consequences of LBW, small head circumference and low Apgar scores simultaneously, capitalizing on a longitudinal design. Our findings are generally consistent with previous literature and extend its scope by highlighting four main findings. First, the number of hospital admissions increased for all three perinatal risk factors-LBW, small head circumference, and low Apgar scores-during the first year of life (0-12 months) and the significant difference by LBW persisted until age 8. Second, each of the three perinatal factors was an independent predictor of neurological, non-neurological, cognitive and language difficulties later on in childhood (ages 4 months-8 years). Third, the difference on various indicators of neurobehavioral impairment between offspring with and without a perinatal factor appear to dissipate on its own over the course of the first few years, however, in adulthood there were modest but significant differences in psychological functioning. Lastly, with the exception of a brief period of time in preschool years, small head circumference and low 5 minute Apgar scores are significant independent predictors for suboptimal functioning, even after controlling for the effect of LBW.

Our findings on the increased risk of hospital admissions in relation to birth weight status were consistent with previously demonstrated links between LBW and hospital admission [32,33]. An anomaly arose in which LBW's association with increased hospital admissions disappeared by age 4 but, reappeared significantly by age 8. Similarly, small head circumference and low Apgar scores were associated with increased hospital admissions in infancy, but the association diminished over time.

There is a large difference between groups for the number of hospitalizations from ages 0 to 12 months because the severity of the same illnesses is intensified for children with LBW. During early infancy, children born with LBW appeared to be more vulnerable, such as by the presence of compromised immune systems, than their normal BW counterparts thus contributing to a substantial difference between groups (p<0.0001). However, for ages 13 to 48 months, those born with LBW who have survived the first 2 years of life might have caught up with their deficits, including their immune functioning. By age 8 the possibly compromised immune systems of children born with LBW might have been highlighted once again when diseases from school exposure may be more likely.

In our study, when the effects of potential confounders, such as sex, race and mother's poverty level during pregnancy, were controlled for, LBW was no longer associated with minor non-neurological abnormalities at 4 months or 1 years of age. Those findings, especially in non-neurological abnormalities among offspring born with LBW, can be explained by malformation in the central nervous system [34] and lower mental development scores [35], which may have contributed towards the persistent non-neurological deficits that were noted. In addition, small head circumference and low Apgar scores were both associated with persistent neurological abnormalities through age 7. While data on changes from therapy and other interventions were not available in this study, more comprehensive testing and evaluations by all therapeutic modalities, which might have been offered at the school setting (i.e., speech, psychology, remedial intervention), could have provided more sensitivity to the deficits and may have allowed for the dissipation of significance.

We found that neurological development at 8 months and 4 years was significantly affected by all of the three perinatal risk factors we examined. Such distinctions between groups persisted in IQ at ages 4 and 7 as well as in achievement scores at age 8. Readers, however, need to be cautious when interpreting the greater magnitude of effects on development as measured by the Bayley Scales. 22 Recent meta-analysis shows that the Bayley Scales on development of very preterm and very LBW children are limited [36]. Additionally, in all but one case, small head circumference and low Apgar scores were associated with problems in reading, spelling and arithmetic abilities. As those perinatal risk factors are relatively easily available for most of the births, considering those indicators to identify the at-risk children may lead to an effective prevention for modifiable academic failures in early primary education. It is also important to point out that not only LBW [37] but also small head circumference and low Apgar scores were strongly associated with abnormalities in language, hearing and speech. Prior research suggests that persistent small head circumference, viewed as an external sign of internal cognitive function or development, is indicative of lower cognitive functioning16 and is often used as a marker of smaller frontal lobes or cortical mass [38,39]. Taken together, our findings suggest that it might not be just LBW, but the quality of intrauterine environment, might be related to poor developmental outcomes in childhood. If so, considering multiple indicators of perinatal risks, not just LBW, we could capture more reliable and valid measure of poor intrauterine environment, and utilizing those extra indicators could help us identify the at-risk children.

Finally, it is important to be reminded that our findings provide evidence that small head circumference was a significant and even stronger predictor of psychological functioning, such as depression, social dysfunction, and somatic symptoms, and low Apgar scores were associated with an increased risk for anxiety. Although growing evidence show that LBW is associated with adult psychiatric disorders [40]. LBW on its own is lacking in terms of its predictive ability of psychological abnormalities in adulthood in the present study. Considering head circumference and Apgar score at birth in conjunction with LBW appear to increase the likelihood of identifying those who would continue to suffer from psychological impairments in adulthood.

Our study has some methodological strength. First, the second generation cohort (N=2,151) was prospectively and systematically followed from birth and studied longitudinally for over 30 years. Our sample came from predominately unprivileged backgrounds of low socioeconomic status with little to no education and a high proportion of ethnic minorities (82.1%). However, they were randomly selected regional (inner-city Baltimore, USA) representatives who sought prenatal care at Johns Hopkins Hospital rather than a clinical sample with either serious psychopathology or medical illness. Nevertheless, we acknowledge that generalizability may be limited. Second, birth weight, Apgar scores, and head circumference were recorded by a research nurse at the time of delivery rather than based on mother's retrospective report. We also acknowledge potential limitations. First, we need to interpret our findings in light of the level of obstetric care in the1960's. Our sample was born in the pre-NICU era and the mortality rate for those born very early and with very LBW was higher than it is currently. Second, although we have adult psychological function data, the participants included in this analysis in adulthood is relatively low. While 75% of adult participants were identified and 67% of the original participants were followed, we were unable to include participants who could have survived had they been born in the modern times, who maybe more likely to have various and more serious impairment. Finally, the concept of special or remedial education, and a realization of the need for early intervention, has also advanced considerably since the late 1960s. It may be that with early intervention and special education programs provided at the preschool level, that some of these results may have ameliorated or disappeared. However, there are also many children who did not survive in the 1960s who would today. They may be more involved and thus more likely to demonstrate long term negative sequellae.

Despite these limitations, the study contributes to the existing literature by providing evidence that 1) examining different perinatal risks, can improve our ability to predict different signs of neuro-cognitive impairments at different times in a life cycle, 2) that small head circumference which does not resolve by age 2 is a risk factor for additional neuro-cognitive impairments, and 3) that low 5 minutes' Apgar scores is a long term predictor of neurological, cognitive, and psychological development. There is ample evidence that early intervention education services can have positive long-term effects most shown for neurological abnormalities and less so for genetic ones [41,42]. Generally, educational programs are funded only when a delay of 25% or 33% can be demonstrated. We believe that our data make a case for the provisions of educational therapeutic services before the delay is marked. The possibility of prevention before delays appear should be considered as part of the early intervention provided to our youngest children for preventative medical, neurological, developmental and psychological measures. Although it is out of the current study's primary focus, future studies should attempt to delineate the precise inter-relationships across perinatal and multiple childhood risk factors, such as poverty, family environment, and peer-relationship. This could facilitate the development of more targeted preventive intervention strategies in high risk cases – parents with suboptimal birth outcomes, especially LBW and small head circumference.

## Figures and Tables

**Table 1:** Number of Hospital Visits at 1 year, 4 Years and 8 Years of Age by Perinatal Problems N=2151

	Birth Weight (BW)			Head circumference			5-min Apgar scores		
	Normal (n=1635)	Low (n=289)		Normal (n=1506)	Small (n=418)		Normal (n=1751)	Low (n=173)	
	Mean (sd)	Mean (sd)	p-value	Mean (sd)	Mean (sd)	p-value	Mean (sd)	Mean (sd)	p-value
**Hospital admissions (N)**									
0 to 12 months	.09 (.18)	.21 (.11)	**p<.0001**	.09 (.02)	.18 (.07)	**p<.0001**	.10 (.01)	.19 (.08)	**p=.005**
13 to 48 months	.16 (.001)	.17 (.01)	p=.77	.16 (.01)	.14 (.02)	p=.43	.16 (.001)	.16 (.01)	p=.89
49 to 96 months	.19 (.01)	.27 (.07)	**p=.02**	.19 (.01)	.24 (.04)	p=.08	.20 (.01)	.25 (.05)	p=.21

NB: Poverty status at the birth of the child, sex of the child and race of the child were statistically controlled for. 7.4% has missing data. There are 10.5% (n=227) missing information

**Table 2:** Neurological and Non-neurological Abnormality at 4 Month, 1 Year and 7 Years of Age by Perinatal Problems

**Neurological assessment**	LBW		Small head circumference		Low Apgar scores	
	AOR (95% CI)	*p*	AOR (95% CI)	*p*	AOR (95% CI)	*p*
4 month assessment	1.0		1.0		1.0	
None (n=1615)						
Suspect (n=509)	**1.8 (1.4-2.4)**	**<.0001**	**1.9 (1.5-2.4)**	**<.0001**	**1.7 (1.2-2.3)**	**.003**
Defnite (n=27) 2151	**4.4 (1.8-10.8)**	**.001**	**3.9 (1.6-9.5)**	**.002**	**4.6 (1.8-12.0)**	**.002**
1 year assessment	1.0		1.0		1.0	
None (n=1828)						
Suspect (n=233)	**2.9 (2.1-4.0)**	**<.0001**	**2.0 (1.5-2.6)**	**<.0001**	**1.6 (1.1-2.5)**	**.02**
Defnite (n=28) 2089	**5.9 (2.7-12.6)**	**.0001**	**4.6 (2.2-10.0)**	**<.0001**	**4.4 (1.9-10.2)**	**.001**
7 year assessment	1.0		1.0		1.0	
None (n=1722)						
Suspect (n=322)	**1.6 (1.2-2.2)**	.**002**	**1.4 (1.1-1.8)**	**.02**	**1.5 (1.0-2.2)**	.**04**
Defnite (n=74) 2118	1.6 (.86-2.9)	.14	**1.9 (1.1-3.2)**	**.02**	**3.2 (1.8-5.7)**	**.0001**
**Non-neurological assessment**	LBW	*p*	Small head circumference		Low Apgar scores	
	AOR (95% CI)		AOR (95% CI)	*p*	AOR (95% CI)	*p*
4 month assessment	1.0		1.0		1.0	
None (n=1509)						
Minor (n=448)	1.2 (.86-1.7)	.27	1.3 (.99-1.7)	.06	1.1 (.73-1.6)	.69
Defnite (n=183) 2140	**1.9 (1.3-2.8)**	**.002**	**1.6 (1.1-2.3)**	**.01**	1.3 (.75-2.1)	.38
1 year assessment	1.0		1.0		1.0	
None (n=1494)						
Minor (n=470)	1.1 (.79-1.6)		1.1 (.79-1.5)		1.1 (.78-1.6)	
Defnite (n=122) 2086	**2.1 (1.2-3.8) .01**	.50	1.1 (.63-1.9) .77	.65	1.2 (.67-2.2) .52	.54
7 year assessment None (n=1579)	1.0		1.0	.21	1.0 1.1 (.79-1.7)	.46
None (n=1579)						
Minor (n=412)	**1.5 (1.0-2.2)**	**.03**	1.2 (.89-1.7)			
Defnite (n=127) 2118	1.2 (.64-2.2)	.62	1.2(.71-2.0)	.50	1.3(.72-2.4)	.39

NB: N may vary due to missing values - 2.9% (n=62) for 1 year and 1.5% (n=33) for the 7 year has missing information on neurological abnormality. 0.5% (n=11), 3.0% (n=65), and 1.5% (n=33) at 4 months, 1 year, and 7 year, respectively, have missing information on non-neurological abnormality.

AOR = adjusted odd ratios. CI = confidence interval. Poverty, sex and race were statistically controlled for.

**Table 3:** Suboptimal Neurological Development at 8 Month and 4 Years of Age by Perinatal Problems

	Birth Weight				Head circumference				5-min Apgar scores		
	Normal	Low			Normal	Small			Normal	Low	
**Suboptimal Neurobehavioral Development**	(n=1764) N (%)	(n=308) N (%)	AOR (95% CI)	p-value	(n=1617) N (%) value	(n=455) value	AOR (95% CI) p-	p-value	(n=1882) N (%)	(n=190) N (%)	AOR (95% CI) p-value
8 Mo Mental^a^	92 (5.2)	78 (25.3)	6.3 (4.4-8.9)	0.0001	92 (5.7)	78 (17.1)	3.6 (2.5-5.0)	0.0002	132 (7.0)	38 (20.0)	3.5 (2.3-5.3)
8 Mo Fine Motor^a^	220 (12.5)	115 (37.3)	4.3 (3.2-5.7)	0.0001	200 (12.4)	135 (29.4)	3.4 (2.6-4.5)	.0002	281 (14.9)	54 (28.4)	2.4 (1.7-3.4)
8 Mo Gross Motor^a^	294 (16.7)	128 (41.6)	3.4 (2.6-4.5)	0.0001	268 (16.6)	154 (33.8)	2.7 (2.1-3.4)	0.0002	359 (19.1)	63 (33.2)	2.1 (1.5-2.9)
8 Mo Social & Emotional^a^	151 (8.6)	56 (18.2)	2.3 (1.6-3.3)	0.0001	136 (8.4)	71 (15.6)	2.0 (1.4-2.7)	0.0002	174 (9.2)	33 (17.4)	2.1 (1.4-3.3)
**Suboptimal Neurobehavioral Development**	(n=1753) N (%)	(n=305) N (%)	AOR (95% CI)	p-value	(n=1603) N (%) value	(n=452) N (%)	AOR (95% CI) p-	p-value	(n=1867) N (%)	(n=190) N (%)	AOR (95% CI) p-value
4 Yr Fine Motor^a^	587 (33.5)	139 (45.6)	1.6 (1.2-2.0)	0.0001	533 (33.3)	193(42.7)	1.4 (1.1-1.8)	0.003	641 (34.3)	85 (44.7)	1.5 (1.1-2.0)
4 Yr Gross Motor^a^	98 (5.6)	41 (13.4)	2.8 (1.8-4.2)	0.0001	93 (5.8)	46 (10.1)	1.9 (1.3-2.8)	0.002	116 (6.2)	23 (12.1)	2.2 (1.4-3.7)
4 Yr Concept Formation^a^	233 (13.3)	52 (17.0)	1.4 (1.0-2.0)	0.03	209 (13.0)	76 (16.8)	1.5 (1.2-2.1)	0.004	252 (13.5)	33 (17.4)	1.4 (.93-2.1)
4 Yr Intelligence^b^	288 (16.4)	80 (26.2)	1.8 (1.3-2.4)	0.0002	260 (16.2)	108(23.9)	1.7 (1.3-2.2)		323 (17.3)	42 (21.1)	1.9 (1.4-2.5)

NB: All tests were administered by child psychologists. N may vary due to missing values (4.6% - 3.4% were missing) AOR = adjusted odd ratios. CI = confidence interval. Poverty, sex and race were statistically controlled for.

a abnormal or suspect;

b borderline or mentally defective.

**Table 4:** Cognitive Functioning at 4 and 7 Years and Academic Functioning at 8 Years by Perinatal Problems

**Cognitive Function**	Mean (sd)	Mean (sd)	F statistics	p-value	Mean (sd)	Mean (sd)	F statistics	p-value	Mean (sd)	Mean (sd)	F statistics	p-value
4 Yr IQ^a^	93.3 (.7)	88.5 (4.1)	F(1, 2048)=31.3	**p<.0001**	93.5 (.93)	89.2 (3.4)	F(1,2048)= 34.4	**p<.0001**	92.9 (.31)	89.4 (3.2)	F(1,2048)=10.9	**p=.001**
7 Yr IQ^b^	92.5 (.64)	88.1 (3.8)	F(1, 2136)=35.6	**p<.0001**	92.6 (.67)	89.4 (2.5)	F(1,2136)= 24.2	**p<.0001**	92.1 (.26)	89.2 (2.7)	F(1,2136)=10.1	**p=.002**
**Learning Function**												
8 Yr WRAT^c^ Reading	31.3 (.46)	28.1 (2.7)	F(1, 2108)=28.7	**p<.0001**	31.2 (.32)	29.7 (1.2)	F(1,2108)= 8.3	**p=.004**	31.0 (.13)	29.5 (1.3)	F(1, 2108)=3.8	**p=.05**
8 Yr WRAT^c^ Spelling	22.5 (.21)	21.0 (1.3)	F(1, 2105)=22.3	**p<.0001**	22.5 (.17)	21.7 (.63)	F(1,2105)= 8.8	**p=.003**	22.4 (.05)	21.8 (.50)	F(1, 2105)=1.9	p=.09
8 Yr WRAT^c^ Arithmetic	19.3 (.25)	17.6 (1.5)	F(1, 2105)=53.2	**p<.0001**	19.2 (.20)	18.3 (.74)	F(1,2105)= 0.6	**p<.0001**	19.1 (.09)	18.1 (.97)	F(1, 2105)=11.9	**p=.001**

a Stanford Benet

b WISCIII

c WRAT = Wide Range Achievement Test. Each area of development at 8 month was evaluated by pediatric neurologists and at 4 years by child psychologists. All tests were administered by child psychologists.

d abnormal or suspect

AOR = adjusted odd ratios. CI = confidence interval. Poverty, sex and race were statistically controlled for.

N may vary due to missing values.

**Table 5:** Abnormality in Language, Hearing, and Speech at 3 Years of Age by Perinatal Problems (N=1956)

	Low Birth Weight		Small Head Circumference		Low 5-min Apgar scores	
	AOR (95% CI)	p-value	AOR (95% CI)	p-value	AOR (95% CI)	p-value
**Language perception**	**1.6 (1.3-2.1)**	**p = .0001**	**2.0 (1.6-2.5)**	**p < .0001**	**1.5 (1.1-2.1)**	**p = .011**
**Language expression**	**1.5 (1.2-2.0)**	**p = .002**	**1.9 (1.5-2.4)**	**p < .0001**	**1.6 (1.2-2.2)**	**p = .003**
**Hearing**	1.2 (.88-1.6)	p = .24	**1.4 (1.1-1.8)**	**p = .02**	1.4 (.92-2.0)	p = .12
**Speech mechanism**	1.2 (.85-1.6)	p = .36	**1.4 (1.1-1.9)**	**p = .006**	1.2 (.80-1.7)	p = .41
**Speech production**	**1.4 (1.1-1.8)**	**p = .02**	**1.3 (1.1-1.7)**	**p = .01**	1.3 (.93-1.8)	p = .13
**Global scoring**	**1.6 (1.2-2.1)**	**p = .001**	**2.0 (1.6-2.5)**	**p < .0001**	1.3 (.96-1.8)	p = .09

NB: N may vary due to missing values (7.6% - 9.1%).

AOR = adjusted odd ratios. CI = confidence interval.

Poverty, sex and race were statistically controlled for.

**Table 6:** Adult Psychological Functioning by Perinatal Problems

	Low Birth Weight		Small Head Circumference		Low 5-min Apgar scores	
	AOR (95% CI)	p-value	AOR (95% CI)	p-value	AOR (95% CI)	p-value
**Depression**	**2.2 (1.0-4.8)**	**p = .05**	**2.6 (1.2-5.2)**	**p = .01**	2.4 (1.0-5.9)	p = .06
**Social Dysfunction**	2.3 (1.3-4.1)	**p = .005**	**2.3 (1.3-3.9)**	**p = .003**	1.9 (1.0-3.9)	p = .095
**Anxiety**	1.2 (.83-1.8)	p = .33	**1.4 (1.0-2.0)**	**p = .03**	1.9 (1.2-3.0)	**p = .004**
**Somatic Symptoms**	1.2 (.80-1.9)	p = .34	**1.5 (1.0-2.2)**	**p = .03**	1.4 (.80-2.4)	p = .25

NB: Of the 2151 birth cohort, 1,540 were located 25 years later. Approximately 91% of the located sample (n=1403) were assessed for their psychological functioning.

AOR = adjusted odd ratios. CI = confidence interval.

Poverty, sex and race were statistically controlled for.
